# Transient Kluver-Bucy Syndrome as a Manifestation of Post-temporal Lobe Seizure: A Rare Case Entity

**DOI:** 10.7759/cureus.31696

**Published:** 2022-11-20

**Authors:** Nicole Latchminarine, Emad A Wahashi, Benedict Amalraj, Ahmed Abubakr

**Affiliations:** 1 Internal Medicine, St. Joseph Mercy Oakland Hospital, Pontiac, USA

**Keywords:** brain injury and seizure, amygdala, kluver-bucy syndrome, transient kbs, post-ictal, temporal lobe seizure, neuropsychiatric manifestations

## Abstract

Following epileptic seizures, patients can subsequently experience a post-ictal state characterized by disorienting symptoms, such as confusion, drowsiness, hypertension, headache, and nausea, rather than neurobehavioral sequelae.

We report the case of a 64-year-old male with unilateral left temporal lobe injury, who presented with post-ictal transient Kluver-Bucy Syndrome symptomatology following a complex partial seizure. Brain magnetic resonance imaging revealed encephalomalacia of the left temporo-parieto-occipital region from a previous infarct, and his symptoms resolved following the administration of antiepileptic medications. Therefore, transient Kluver-Bucy Syndrome can follow unilateral temporal lobe injury and should be suspected in patients who fit the clinical criteria, even in the absence of classic bilateral temporal lobe damage on imaging.

## Introduction

Kluver-Bucy Syndrome (KBS) is a rare and complex neuro-behavioral disorder that is classically associated with injury to the bilateral temporal lobes, specifically involving the amygdala and hippocampus [1]. It was first described in 1937 as an experimental neurobehavioral syndrome in Rhesus monkeys following bilateral temporal lobectomy [2-5]. Terzian and Ore reported the first human description of KBS in 1955 after a bilateral temporal lobectomy was performed on a man for seizure treatment [5]. Subsequently, in 1975, a case of KBS was observed in a patient with bilateral temporal lobe lesions resulting from meningoencephalitis [4].

The presentation of KBS can vary; however, the neurological indicators typically include visual agnosia and distractibility, hyperorality, hyperphagia, hypersexuality, placidity, emotional and memory impairment, and hypermetamorphosis. Patients with classic KBS demonstrate all of these symptoms, whereas patients with partial KBS exhibit three or more. KBS is diagnosed clinically based on the presence of characteristic symptoms, however, brain magnetic resonance imaging (MRI) typically confirms the diagnosis by demonstrating bilateral temporal lobe injury [5]. KBS is widely known to be associated with a diverse range of etiologies, including herpes simplex virus-1 (HSV-1) encephalitis, tubercular meningitis, bilateral temporal lobectomy, traumatic brain injury, bilateral temporal lobe infarction, paraneoplastic limbic encephalitis, Pick’s and Alzheimer’s dementia [2-3,6]. While KBS has been observed most commonly after bilateral temporal lobe mutilation, few cases have been reported in the literature of transient KBS following unilateral injury [7-9]. We report a case of a 64-year-old man with a prior temporal lobe infarct who exhibited symptoms of post-ictal transient KBS following a complex partial seizure.

## Case presentation

A 64-year-old male, with a history of left temporal lobe infarction in June 2018, one prior seizure in September 2018 on levetiracetam, hypertension, and prior alcohol abuse, presented to the emergency department in February 2019 with the chief complaint of altered mental status. Three hours prior to his arrival, the patient complained of a headache and was experiencing formed visual hallucinations of people having intercourse in his living room. Additionally, he vocalized not knowing where he was and that he did not recognize his home. On the way to the hospital, he had a witnessed seizure where he was screaming, biting his tongue, and foaming at the mouth. His body also became very stiff and his eyes rolled back; this lasted for approximately one minute.

Upon arrival at the emergency department, he was hypertensive at 163/90 mmHg and tachycardic at 105 beats/minute. He was unable to recognize his family and was vocalizing random words and screaming. He also experienced hyperorality with tongue and finger biting to make them bleed, inappropriate sexual behavior, and was speaking about sexual intercourse and erections. The patient did not exhibit any stiffness or jerking of his body at this time and did not have further seizure activity.

On examination, he was awake and conscious but very confused, non-oriented, and was experiencing formed visual hallucinations. He had difficulty following commands and had a forced rightward gaze that did not cross the midline. There was significant right upper extremity weakness, and he was unable to hold it against gravity and complained of a headache. His home medications at the time were Aspirin 81 mg, atorvastatin 80 mg, levetiracetam 500 mg twice daily, lisinopril 20 mg twice daily, and amlodipine 10 mg. Per the patient’s wife, he was able to perform activities of daily living at baseline, however, she was unsure if the patient had been compliant with his levetiracetam.

Several laboratory investigations were conducted, including a complete blood count, coagulation profile, electrolyte panel, and troponins. The patient’s labs were significant for hyponatremia, hypochloremia, and hypocapnia, which was consistent with his recent seizure. The remainder of his labs were within normal limits (Tables 1-2). His urine drug screen was negative. CSF analysis was not completed on the patient, and neither was a plasma levetiracetam level.

**Table 1:** Initial basic metabolic panel and troponin

Basic Metabolic Panel	Value	Reference Range
Sodium	128 mEq/L	135-144 mEq/L
Potassium	4 mEq/L	3.5-5.3 mEq/L
Chloride	92 mEq/L	98-107 mEq/L
Carbon Dioxide	18 mEq/L	21-31 mEq/L
Glucose	153 mg/dL	70-90 mg/dL
Blood Urea Nitrogen	15 mg/dL	7-25 mg/dL
Creatinine	0.97 mg/dL	0.60-1.20 mg/dL
Glomerular Filtration Rate Non-African American	94 mL/min	>60 mL/min
Calcium Total	9 mg/dL	8.6-10.3 mg/dL
Troponin I	<0.03 ng/mL	0-0.04 ng/mL

**Table 2:** Initial complete blood count and coagulation study

Complete Blood Count	Value	Reference Range
White Blood Cells	7.9 thousand/mcL	3.7-11.0 thousand/mcL
Red Blood Cell Count	5.09 million/mcL	3.80-5.20 million/mcL
Hemoglobin	13.7 gm/dL	12.0-16.0 gm/dL
Hematocrit	42.6%	35.0-46.0%
Mean Corpuscular Volume	84 FL	80-99 FL
Mean Corpuscular Hemoglobin Concentration	32.2%	29.0-37.5%
Red Cell Distribution Width	13.3%	12.0-15.0%
Platelet Count	302 thousand/mcL	140-440 thousand/mcL
Prothrombin Time	11.6 seconds	9.5-12.5 seconds
International Normalized Ratio	1	2.0-3.0
Activated Partial Thromboplastin Time	26.7 seconds	30.0-40.0 seconds

**Figure 1:**
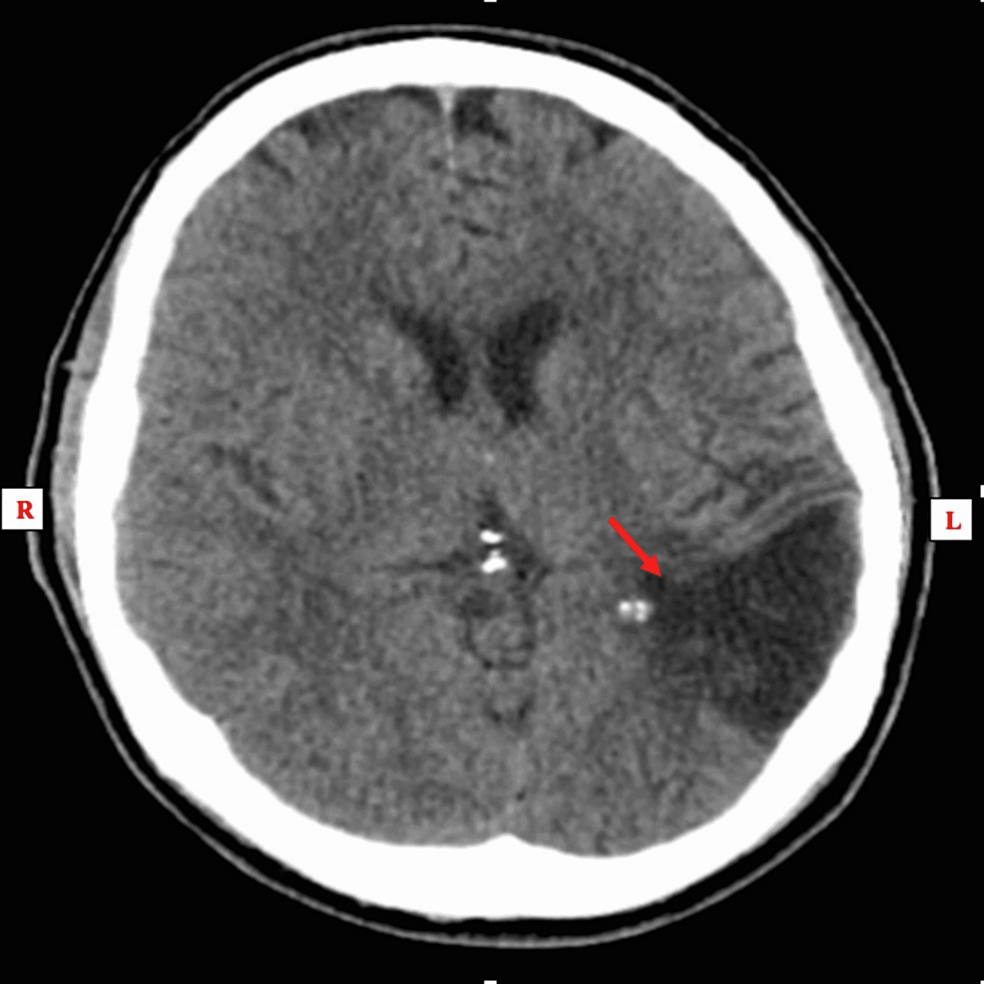
CT head without contrast (axial view); re-demonstration of encephalomalacia in the left temporoparietal lobe from the prior infarct

**Figure 2:**
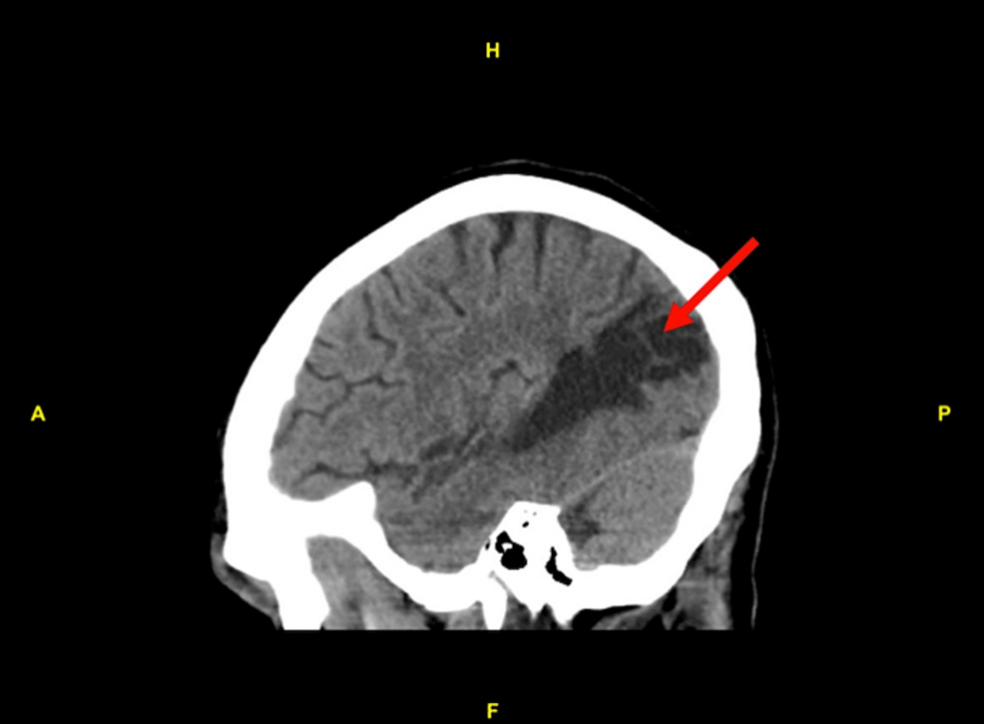
CT head without contrast (sagittal view); re-demonstration of encephalomalacia in the left temporoparietal lobe from the prior infarct

**Figure 3:**
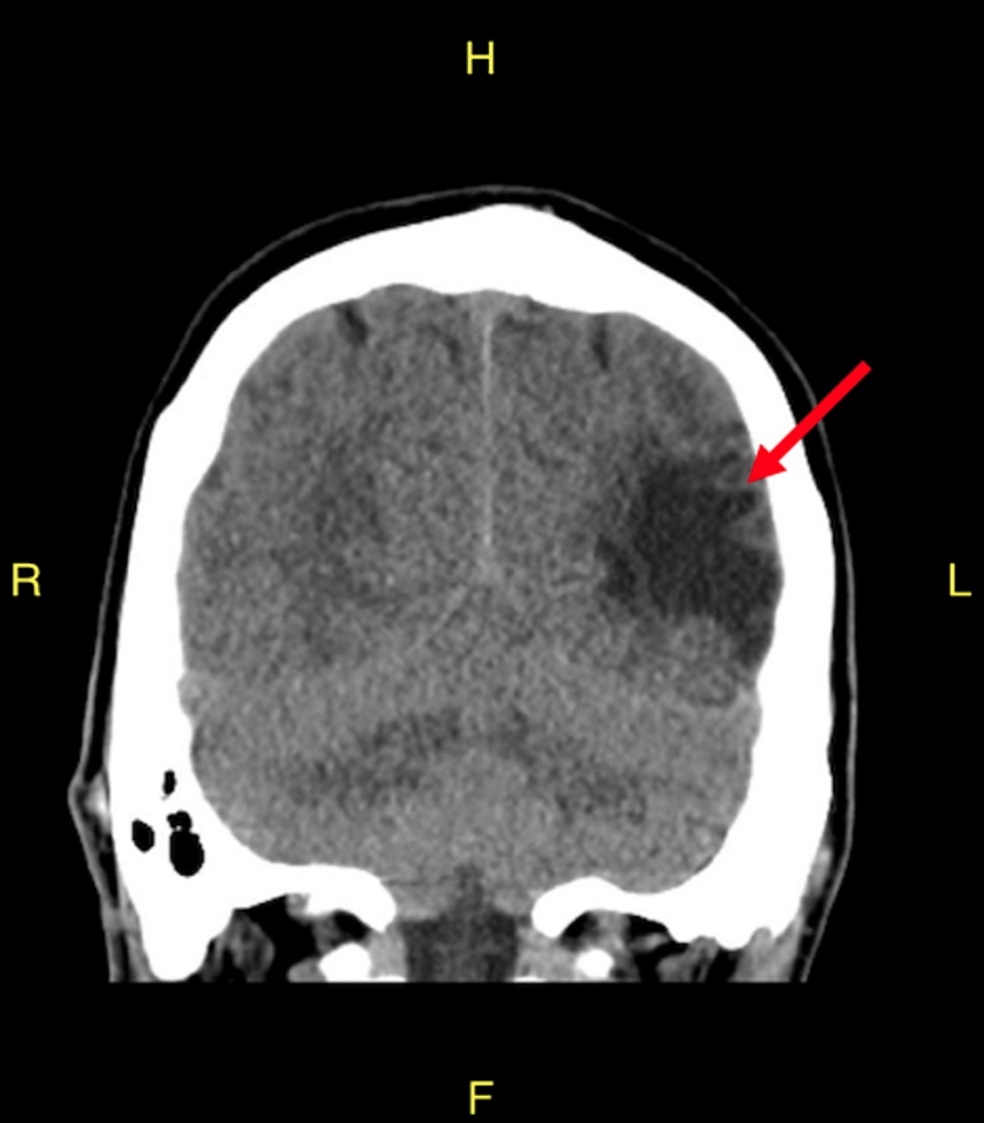
CT head without contrast (coronal view); re-demonstration of encephalomalacia in the left temporoparietal lobe from prior infarct

An electrocardiogram (EKG) was done and was unremarkable. An emergent head computed tomography (CT) scan without contrast re-demonstrated an old left temporoparietal lobe encephalomalacia from his prior infarct in June 2018; there was no evidence of acute intracranial hemorrhage (Figures 1-3).

The patient had an electroencephalogram (EEG) done five months prior (September 2018) following his first seizure episode. Preceding that seizure episode, the patient was behaving strangely and was not at his baseline. He was noted to be standing in the kitchen alone in the middle of the night, cooking food for his family. When the seizure occurred, his daughter heard a loud noise from the bathroom and when she opened the door, she found the patient lying on the floor, unresponsive to his name, and he was physically shaking and foaming and drooling at his mouth. This episode lasted for five to seven minutes. Upon return to consciousness, he had difficulty articulating his speech. The EEG from that admission indicated focal slowing and an asymmetric background was seen over the left hemisphere, which was consistent with structural and physiologic dysfunction. These waveforms were considered non-epileptiform in nature. Unlike the second seizure episode that is being described in this case, the patient exhibited no KBS-like symptoms preceding and during the post-ictal phase.

**Figure 4:**
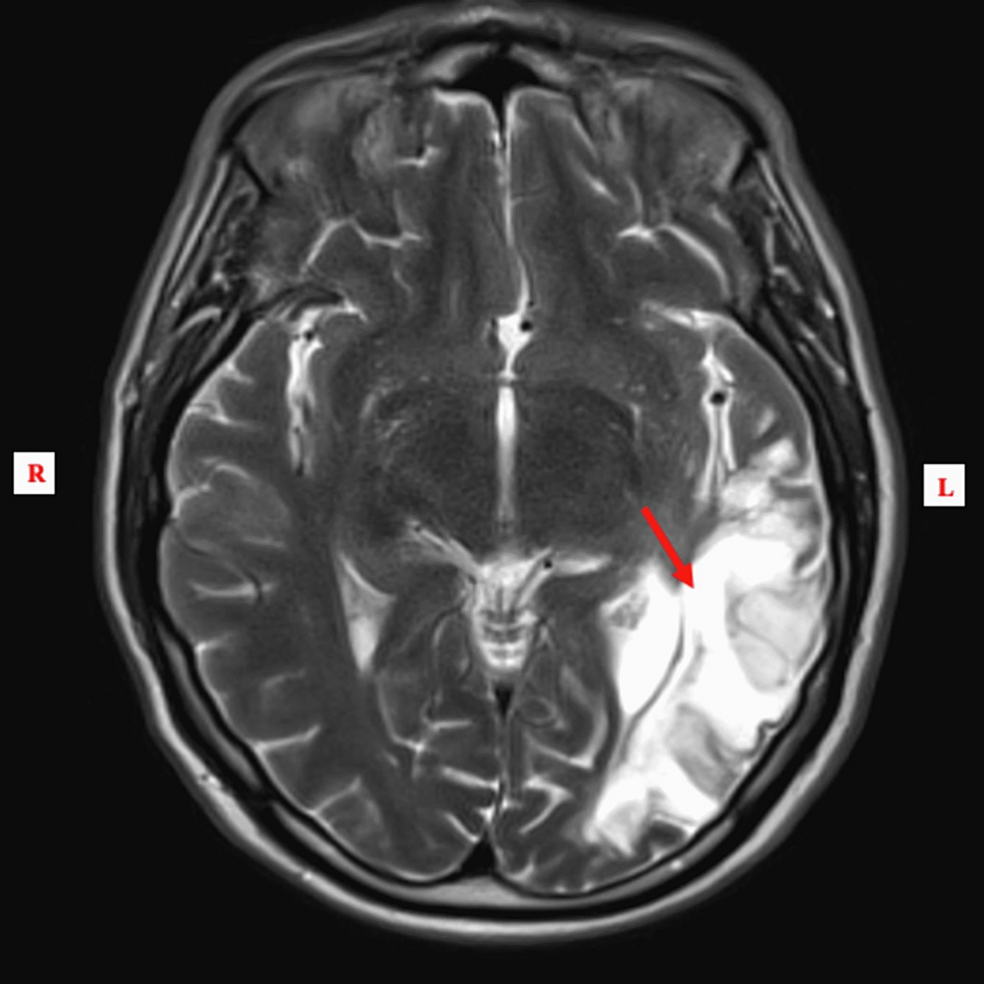
MRI with contrast (axial view) notable for a large area of encephalomalacia in the left temporoparietooccipital region

**Figure 5:**
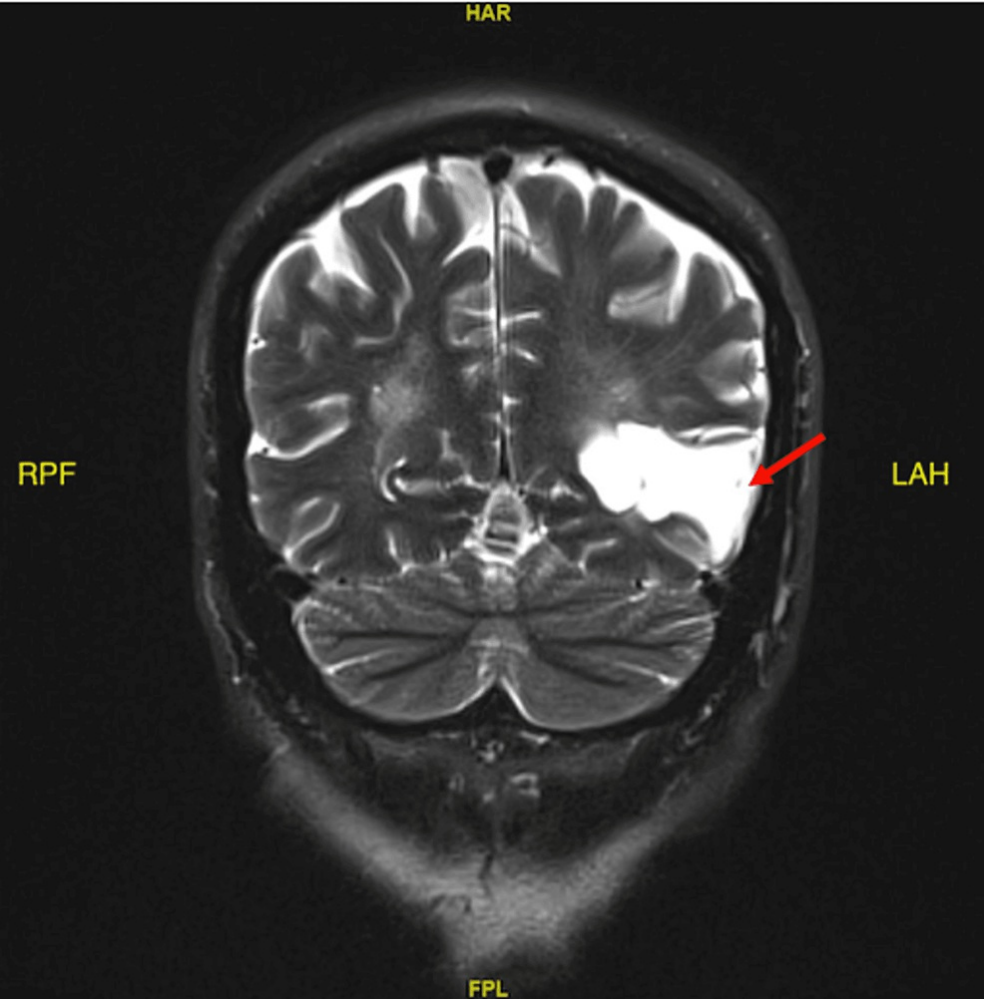
MRI brain with contrast (coronal view), indicating large area encephalomalacia in the left temporoparietooccipital region

During this admission however, his EEG was normal, as there was no evidence of epileptiform discharges, paroxysmal activity, or lateralizing features in the tracing. Neurology was consulted and recommended a follow-up MRI of the brain with contrast, which revealed a large area of encephalomalacia from remote infarction in the left temporo-occipital region (Figures 4-5). T2/fluid-attenuated inversion recovery (FLAIR) showed no acute ischemic changes. These findings were consistent with the patient’s history of left temporal lobe infarction in June 2018. Additionally, psychiatry evaluated the patient and determined that his presentation was secondary to medical reasons.

A complex-partial temporal lobe seizure was considered to be the root cause of his symptoms. The patient was given a loading dose of lacosamide (400mg) and later continued levetiracetam 500 mg twice daily while bridging with lacosamide. Levetiracetam was gradually tapered. Shortly after the loading dose of lacosamide, the patient’s post-ictal confusion and KBS symptoms resolved. He became more alert and awake, and no longer had a gaze deviation, though he did have some residual right upper extremity weakness, which was thought to be due to Todd’s paralysis. Of note, his symptoms were transient and limited to the post-ictal period.

## Discussion

KBS is a rare and complex neuropsychiatric condition classically associated with bilateral injury to the medial temporal lobes [1,10-11]. Clinically, hyperorality, placidity, and dietary changes are the most commonly occurring symptoms of KBS [12]. Most cases that have been reported of KBS in humans were associated with progressive neurological pathologies and did not present transiently. Specifically, in the literature, there are few published cases of KBS limited to the post-ictal period. The patient described in this report exhibited multiple behavioral symptoms associated with KBS, including self-mutilation, hyperorality, confusion, visual agnosia, sexual preoccupation, and hypermetamorphosis following a complex-partial seizure, and this was limited to the post-ictal period. The patient had a left temporal lobe injury secondary to a prior cerebral infarction. Initial CT upon presentation revealed no bleeding or indication of a new stroke, and thus he was evaluated for a post-ictal phenomenon. His bizarre behavior fit the clinical presentation of KBS, as hyperorality, hypersexuality, and genital automatisms have been reported in patients with bilateral temporal lobe epileptic foci or unilateral left-sided foci. The follow-up brain MRI displayed a large area of encephalomalacia from his previous infarct of the left temporal-occipital region. This regional scarring was suspected to be the epileptogenic focus of the breakthrough seizure, as he was non-compliant with his antiepileptic medications. Subsequently, after the resumption of the patient’s medication, his bizarre symptoms and behavior resolved, thus confirming the likelihood of transient post-ictal KBS.

Similarly, Anson and Kuhlman reported a case of recurrent KBS behavior in a 24-year-old female, five years after a left medial temporal lobectomy secondary to status epilepticus refractory to maximal medical therapy. In their reported case, the patient was lost to follow-up after the lobectomy and experienced seizures when her antiepileptic medication ran out. In the post-ictal period, she exhibited symptoms of hypersexuality and placidity. Her behavior was limited to the post-ictal period and was normal between seizure episodes [13].

It is known that the majority of complex-partial seizures, also known as "focal impaired awareness seizures" originate from the temporal lobe. It has been noted in the literature that 40-80% of patients with temporal lobe epilepsies present with oral and manual automatisms and changes in sexual behavior during the ictal phase of temporal lobe seizures [14]. This manifestation, however, does not seem to account for the altered behavioral syndrome exhibited in our case, as the patient’s symptoms were limited to the post-ictal period. It is plausible that in the post-ictal period, the non-infarcted right temporal lobe temporarily misfunctioned, thus eliciting a bilateral temporal lobe injury presentation (KBS). Upon resumption of the patient’s home antiepileptic medications, his right temporal lobe function may have improved, and thus the KBS manifestations resolved. Perhaps the transient loss of function of the right temporal lobe in conjunction with the prior left temporal lobe injury created a functional bilateral temporal lobe injury or lobectomy-like presentation, similar to the known neuropathological mechanism of KBS [12-13].

This case illustrates that despite being rare, KBS can in fact present acutely and transiently in patients with unilateral brain injury. The clinical diagnosis can be made based on symptomatic presentation and should be considered a plausible etiology even in the absence of traditional bilateral findings on brain imaging.

## Conclusions

Patients with KBS characteristically have bilateral damage to the temporal lobes on brain MRI. Transient KBS secondary to complex-partial lobe seizures should be suspected in patients who fit the clinical criteria even in the absence of classical MRI findings. Our patient had post-ictal transient KBS symptoms preceding and following a breakthrough seizure from an epileptogenic focus in the left temporal lobe, likely resulting from a prior temporal lobe infarct. His symptoms resolved subsequently after resuming and adjusting the antiepileptic medications. Notably, transient KBS is a rare presentation of temporal lobe seizures and very few cases have been reported in the literature to date.
